# Cellular Senescence in Physiological and Pathological Processes

**DOI:** 10.3390/ijms232113342

**Published:** 2022-11-01

**Authors:** Mauro Finicelli, Gianfranco Peluso, Tiziana Squillaro

**Affiliations:** 1 Research Institute on Terrestrial Ecosystems (IRET), National Research Council of Italy (CNR), Via Pietro Castellino 111, 80131 Naples, Italy; 2 Faculty of Medicine and Surgery, Saint Camillus International University of Health Sciences, Via di Sant’Alessandro 8, 00131 Rome, Italy; 3 Department of Experimental Medicine, University of Campania “Luigi Vanvitelli”, Via Santa Maria di Costantinopoli 16, 80138 Naples, Italy

This Special Issue aims to address the impact of cellular senescence on human biology, looking at both physiological and pathological processes. Senescence is the permanent cell cycle arrest that occurs in response to extracellular or intracellular stress. It determines the loss of cellular functions over time. In addition, numerous studies correlate cellular senescence to aging, as it limits the proliferation of damaged cells largely contributing to the reduction in tissue functions and renewal [1]. In this regard, one of the manuscripts submitted for this Special Issue focuses on the role of senescence in skin aging. Skin aging and senescence has become a popular topic given its occurrence is due to the combinatory effect of intrinsic factors (i.e., chronological aging) and external cues, such as environmental stresses (e.g., sun exposure and air pollution) [2,3,4]. Zorina et al. focused on the detrimental effect of senescence in fibroblasts: the main cellular population of the dermis. Indeed, the accumulation of senescent dermal fibroblast can contribute to the depletion of the stem/progenitor cell pool in a paracrine way through the senescence-associated secretory phenotype (SASP). This could, in turn, impair the regenerative ability of the dermal tissue and predisposing it to aging and other consequential diseases [5,6,7]. A body of literature has sustained the role of senescent cells secreted by SASP proteins and extracellular vesicles (exosomes) to carry out several functions such as sensitizing normal neighboring cells to senesce, reinforcing the senescence process through autocrine signaling, inducing tissue remodeling and repair, and promoting wound healing and immune cell recruitment [1,8].

Cellular senescence is also involved in cancer biology in a dual way: (i) by promoting protective anticancer mechanisms resulting in tumor growth arrest; (ii) or, paradoxically, by inducing cancer onset through the persistence of senescent cells in tissues. With regards to this topic, the review of Salazar-Terreros and Vernot gives an interesting hint about the effect of senescent cells in influencing the microenvironment which they live in [9]. The authors focused on the alteration of hematopoietic stem cells niche in bone marrow (BM) due to senescent mesenchymal stromal cells (MSCs), given MSCs are the major and relevant BM components. During aging, the phenotype and function of these cells change, triggering a senescent state that, in turn, impairs the cellular biology and aspects such as metabolism and secretome composition [9,10,11]. This could lead to a decrease in the immunomodulatory properties of MSCs and the promotion of proliferation and migration of leukemic cells [9,11,12]. This could support a microenvironment enhancing malignant cell survival. Given the lack of suitable in vitro and in vivo models able to reflect the complexity of the events triggered in bone marrow, the authors provide a focus on in leukemic niche modeling with a special overview on the contribution of senescent MSC on leukemia progression.

Another key aspect linking senescence to cancer is the concept of the “therapy-induced senescence”. This is a process mainly associated with tumor response to radiotherapies in cancer cell, due to the failure of ionizing radiation (IR) in bypassing the threshold needed to induce apoptosis in cancer cells. This is an intriguing topic that has been argued by Russo et al. in our Special Issue. The authors investigated the ability of different senolytics, including two natural flavonoids (quercetin and fisetin), to increase IR sensitivity in two radio-resistant cellular sub-populations derived from human osteosarcoma and colon adenocarcinoma cell lines (SAOS and HT29, respectively). Their data demonstrated that senolytic agents were able to sensitize SAOS400 and HT500 (radio-resistant cellular sub-populations derived from parental SAOS and HT29) to cell death induced by γ irradiation. Of note, the combinatory effect of these molecules with γ irradiation led to a decrease in the levels of p16^INK4^ and p21^CIP1^, along with synergistically induced increase in cell death compared to radiation mono-alone treatments [13]. These results pave the way for further studies dissecting the potential role of senolytics as adjuvant agents in cancer therapy and allow us to explore another intriguing research topic on senescence. Senotherapeutics make up a novel class of drugs under scrutiny for delaying aging and associated diseases. The natural phytochemicals, although less potent when compared to synthetic molecules, represent a promising therapeutic approach. They have low toxicity and elicit less adverse effects; thus, they are suitable candidates for transition in clinical settings. Among them, polyphenols commonly present in fruit and vegetables, including naringenin, hesperidin, quercetin, fisetin, rutin, apigenin, luteolin, and epigallocatechin gallate, have showed antisenescence effects [8,14,15,16].

This evidence pairs those showing that the pharmacological ablation of senescent cells improves longevity and promotes health span [1,8,17]. On this regard, the review of Liu focuses on the contribution of cellular senescence on the onset of Alzheimer’s disease (AD), an aging-related neurodegenerative disease and a major cause of dementia in the elderly [18]. Findings from the brains of AD patients and AD animal models revealed the presence of senescent astrocytes, microglia, endothelial cells, and neurons. The limitation of senescence, either via genetical or pharmacological approaches, resulted in the amelioration of β-amyloid (Aβ) peptide and tau-protein-induced neuropathologies, as well as an improvement in the memories of AD model mice [18,19,20,21,22,23]. Thus, an analysis of the recent literature allowed the authors to sustain that senescence reduction could provide effective benefits for the treatment of age-related diseases, such as AD [18].

Despite the accumulated knowledge from the last decade has allowed us to address many questions for senescence, some answers still arose. So, further research is strongly needed to provide further insight on the matter, as the creation of appropriate models facilitates suitable and reproducible findings. On this aspect, Greasymuchuk et al. published an intriguing article on this Special Issue about the set-up of a H_2_O_2_-induced senescence model of stress-induced premature senescence (SISP). The authors aimed to establish a reliable model of cellular senescence in vitro, given that this field of research is widespread and needs robust and reproducible models. Authors tested three-step and one-step H_2_O_2_-induced senescence models of SIPS and showed that they both were able to trigger similar aging features, as in cells with replicative senescence. Indeed, they found that treatments induced an increase in β-Gal staining, in p21 expression levels, as well as a deregulation in the expression profiles of cell cycle regulator factors, an alteration in extracellular matrix composition, and poor viability. Of note, they speculated that the one-step senescence model performed the best, given that it evoked a significant increase in senescent biomarkers, an alteration in metabolism, and reduced functionality on cultured fibroblasts. They also provided intriguing speculations on the feasibility of this in vitro senescence model for anti-aging compounds testing [2]. On the other hand, Ain et al. published the setting-up of a protocol for the isolation of viable neural cells from mature central nervous system (CNS). Indeed, age-related changes may alter biophysical and biochemical properties of CNS tissues, and thus may interfere with the efficiency of common isolation techniques [24].
